# Ventricule droit à double chambre (VDDC) isolé: à propos d'un cas au CNHU, Cotonou, Bénin

**DOI:** 10.11604/pamj.2017.27.7.11115

**Published:** 2017-05-02

**Authors:** Philippe Mahouna Adjagba, Arnaud Sonou, Léhila Bagnan Tossa, Léopold Codjo, Murielle Hounkponou, Salimatou Assani Moutaïrou, Yasmine Eyissè Kpossou, Latif Moussé, Yessoufou Tchabi, Jeanne Vehounkpé Sacca, Martin Dèdonougbo Houénassi

**Affiliations:** 1 Service de Cardiologie, CNHU-HKM, Cotonou, Université d'Abomey-Calavi (UAC), Bénin; 2 Centre Hospitalier Universitaire Départemental de l'Ouémé et du Plateau, Porto-Novo, Université d'Abomey-Calavi (UAC), Bénin; 3 Service de Pédiatrie et de Génétique Médicale, CNHU-HKM, Cotonou, Université d'Abomey-Calavi (UAC); 4 Centre Hospitalier Universitaire Départemental du Borgou et de l'Alibori, Parakou, Université de Parakou (UP), Bénin; 5 Polyclinique Atinkanmè, Cotonou, Bénin

**Keywords:** VDDC, adolescente, syncope, échocardiographie, chirurgie, DCRV, teenager, syncope, echocardiography, surgery

## Abstract

Le ventricule droit à double chambre (VDDC) est une malformation cardiaque rare dans laquelle le ventricule droit est divisé en deux chambres par une bande musculaire anormale. Il est associé dans 80 à 90% à d'autres malformations. L'expression clinique est variée et dépend de l'importance de l'obstruction intraventriculaire. Nous rapportons l'observation d'une adolescente de 16 ans, présentant une forme isolée de VDDC révélée par des syncopes à répétition. Le diagnostic est fait par l'échocardiographie Döppler. La résection chirurgicale de la bande musculaire anormale a été faite avec succès.

## Abstract (en)

Double-chambered right ventricle (DCRV) is a very rare cardiac defect in which the right ventricle is divided by anomalous muscle bundle into two chambers. It is associated with other malformations in 80-90% of cases. Clinical presentation varies and depends on the extent of the intraventricular obstruction. We here report the case of a 16-year old teenager with isolated DCRV revealed by recurrent syncopes. The diagnosis was made using Doppler echocardiography. The patient underwent successful surgical resection of the abnormal muscle band.

## Introduction

Le ventricule droit à double chambre (VDDC) est une cardiopathie congénitale rare dans laquelle le ventricule droit est divisé en deux chambres par une bande musculaire anormale [1], son incidence varie entre 0,5 et 2% [2]. La plupart des porteurs de VDDC sont diagnostiqués dans l'enfance ou à l'adolescence, avant l'âge de 20 ans, cependant des présentations à l'âge adulte peuvent être rencontrées. La symptomatologie clinique est variée et dépend de l'importance de l'obstruction intraventriculaire. Le VDDC est associé dans 80 à 90% des cas à d'autres malformations congénitales. Le traitement est chirurgical avec un excellent pronostic à long terme. Nous rapportons l'observation d'une patiente diagnostiquée pour VDDC et prise en charge chirurgicalement.

## Patient et observation

**Figure 1:**
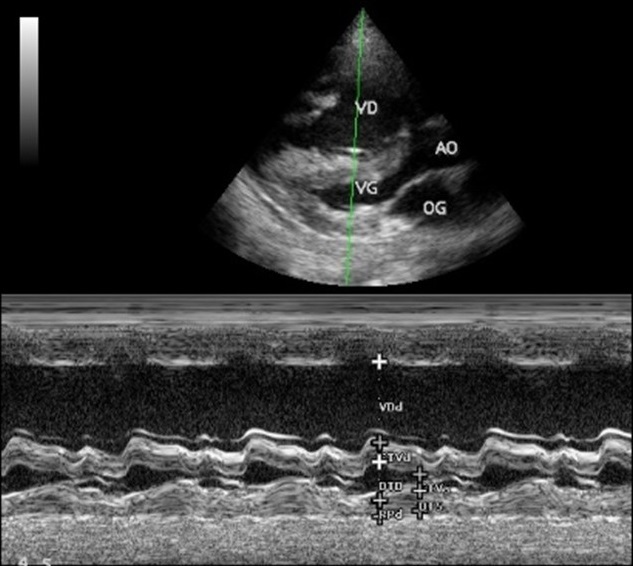
Pré-opératoire: coupe parasternale grand axe, mode bidimensionnel (image en haut) et mode TM (image en bas); Ventricule droit (VD) dilaté avec paroi antérieure épaissie; Ventricule gauche (VG) apparaît relativement petit avec une bonne fonction systolique; AO: aorte; OG: oreillette gauche

**Figure 2:**
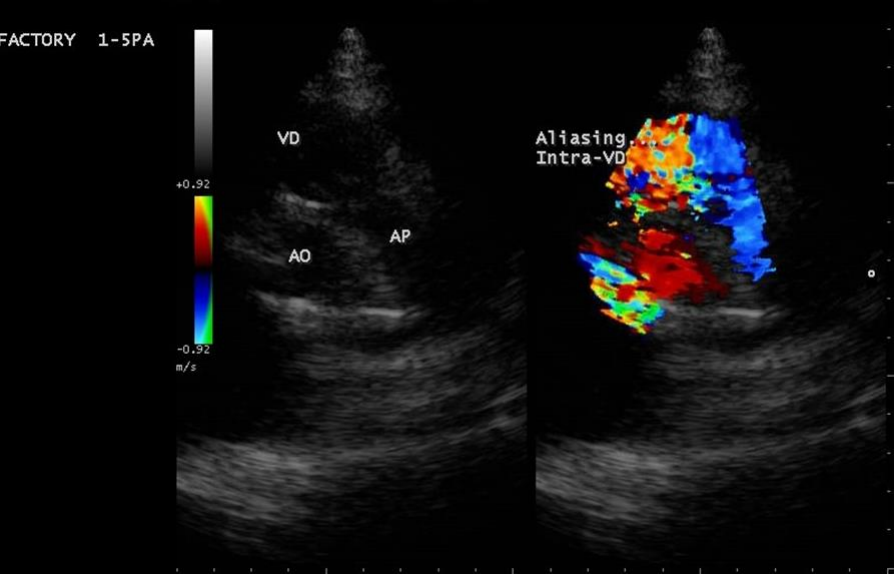
Pré-opératoire: incidence parasternale petit axe, 2D (gauche) et Döppler couleur (droite) avec aliasing témoignant d'une obstruction intra-VD; Ao: aorte; AP: artère pulmonaire

**Figure 3:**
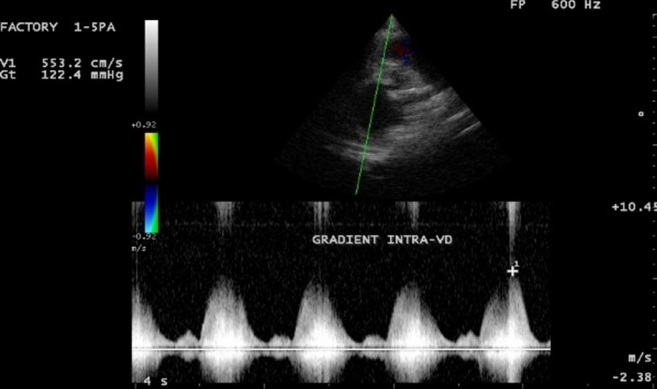
Pré-opératoire: incidence para-sternale petit axe, Döppler continu avec flux intra ventriculaire avec gradient de 122 mmHg

**Figure 4:**
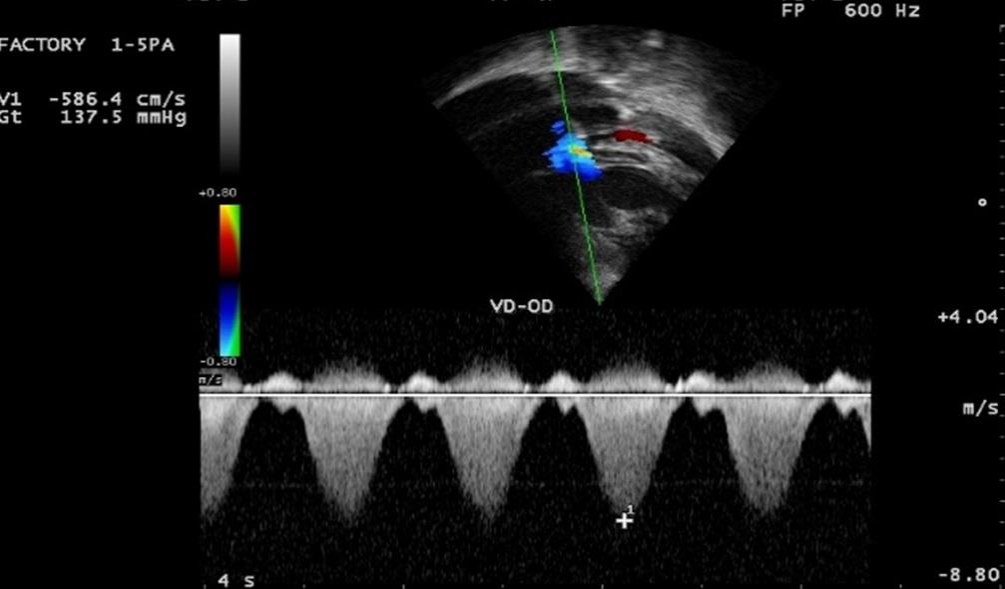
Pré-opératoire: Doppler continu sur le flux d'Insuffisance tricuspide (IT, en bleu); Gradient VD-OD de 137 mmHg

Il s'agit d'une adolescente de race noire, âgée de 16 ans, reçue en consultation pour dyspnée d'effort et deux épisodes de syncope d'effort. Elle avait rapporté une dyspnée d'effort stade II-III de la NYHA évoluant de façon intermittente depuis l'enfance. On retrouve une majoration de la dyspnée deux semaines avant son admission, une fatigabilité à l'effort, avec deux épisodes de syncope survenue à l'effort. Ses antécédents personnels médicaux, chirurgicaux et familiaux étaient sans particularité. L'état général était bon. Il n'y avait pas de cyanose. Les constances vitales étaient normales avec une pression artérielle à 134/96 mmHg, une fréquence cardiaque à 90/mn, une SaO2 à 96 %, un poids de 39 kg, une taille de 1,50 m avec un indice de masse corporelle (IMC) de 17,33kg/m^2^. L'examen cardiovasculaire retrouvait un rythme régulier, un souffle systolique rude d'intensité 5/6 maximum en parasternal gauche et irradiant sur tout le précordium et dans le dos. Il n'y avait pas de signe d'insuffisance cardiaque. **L'électrocardiogramme (ECG)** s'inscrivait en rythme sinusal avec quelques extrasystoles ventriculaires monomorphes isolées. L'axe du QRS était à +120 degrés et on retrouvait des signes de surcharge cavitaire droite. **Le télécœur** mettait en évidence une cardiomégalie avec index cardio-thoracique à 0,79 et une hypovascularisation pulmonaire modérée. **L'échocardiographie Doppler** (Figure 1, Figure 2, Figure 3, Figure 4) retrouvait la présence d'une bande musculaire intraventriculaire droite responsable d'une partition du ventricule droite en deux chambres avec pression ventriculaire droite élevée dans la chambre d'amont, estimée en Doppler continu à 122 mmHg de pic. Le ventricule droit était dilaté et hypertrophié avec une bonne contractilité. La voie pulmonaire était normale. La pression ventriculaire droite (PVD) évaluée sur le flux d'insuffisance tricuspide était supra systémique et estimée à 147 mmHg. La courbure septale était de type III en systole et type II en diastole. Il n'y avait pas de communication intraventriculaire.

**Figure 5:**
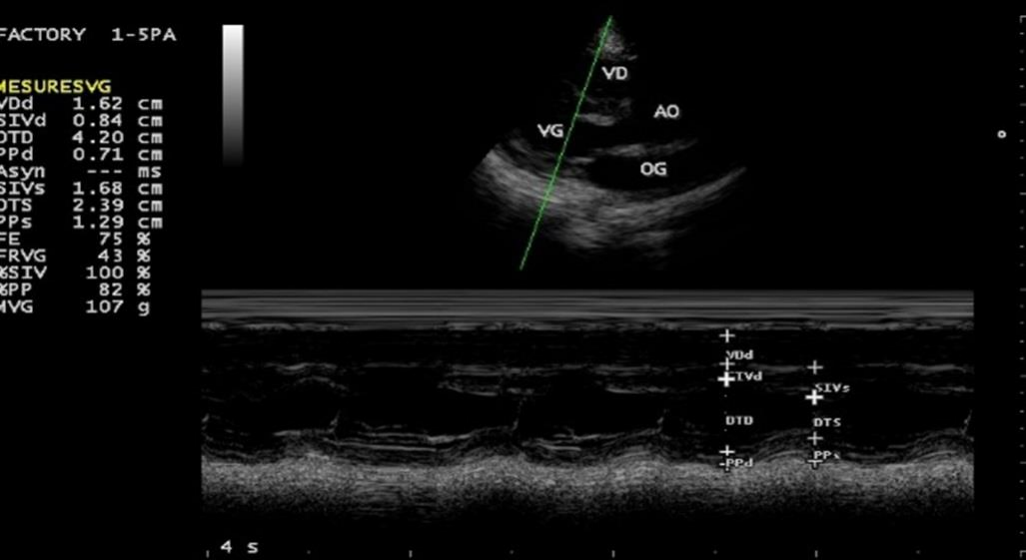
Post-opératoire (3 mois): coupe parasternale grand axe, mode bidimensionnel (image en haut) et mode TM (image en bas), VD de taille normale; VG de taille normale avec une bonne fonction systolique

**Figure 6:**
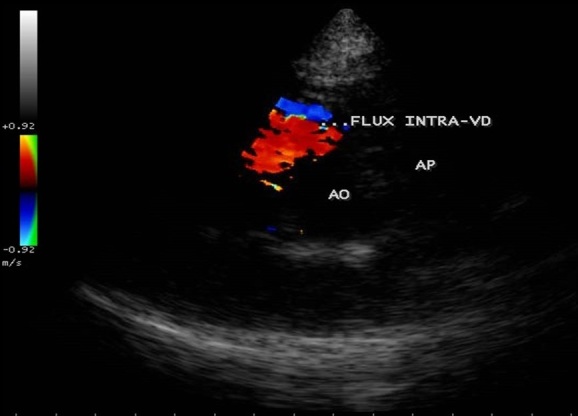
Post-opératoire (3 mois): incidence parasternale petit axe, Döppler couleur avec flux laminaire témoignant d'une absence d'obstruction intra-VD

**Figure 7:**
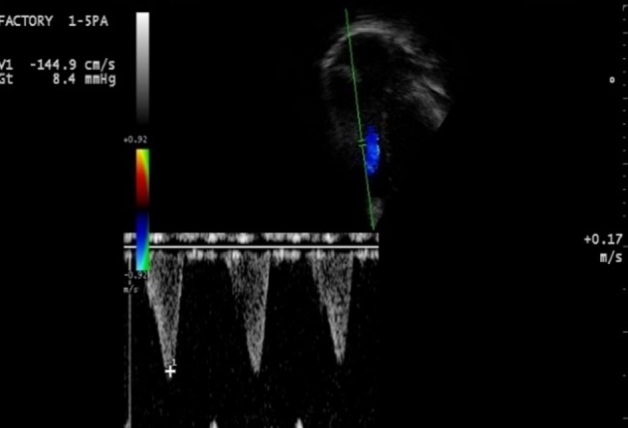
Post-opératoire (3 mois): incidence apicale 4 cavités, Flux Döppler pulsé intra-ventriculaire droite, gradient de pic à 8,4 mmHg

**L'angioscanner artériel pulmonaire** retrouvait une belle voie pulmonaire et une importante dilatation des cavités cardiaques droites. Le traitement institué était du propanolol à la dose de 3 mg/kg/j en 3 prises. La cure chirurgicale a consisté en la résection de la bande musculaire intra-ventriculaire droite par sternotomie médiane sous circulation extracorporelle (durée 39 mm). Par une ouverture de l'infundibulum, il a été mis en évidence un rétrécissement très serré à 1cm de la valve pulmonaire avec une bague fibreuse de 4 mm de diamètre où passait tout le flux du cœur droit. Il n'existe aucune communication interventriculaire. La PVD était supra systémique. Une résection abondante de muscles et de bandelettes musculaires sténosantes a été réalisée. En post intervention la PVD était à 20% de la pression systémique témoignant d'un bon résultat immédiat. Les suites opératoires étaient simples avec extubation précoce et sortie des soins intensifs 24 heures après la chirurgie. A l'évaluation clinique à 3 mois puis à 5 mois post-opératoire, la patiente était asymptomatique sous propanolol 3 mg/kg/j en 3 prises. Elle avait complètement repris ses activités sociales. L'examen physique était normal sans souffle à l'auscultation cardiaque. L'électrocardiogramme s'inscrit en rythme en sinusal avec un bloc de branche droit complet. L'échocardiographie Döppler (Figure 5, Figure 6, Figure 7) mettait en évidence une régression notable de la dilatation ventriculaire droite, une absence de gradient intraventriculaire droit significatif. La voie pulmonaire était libre de sténose et de fuite significative. La courbure septale était normale en diastole et en systole (témoignant d'une PVD infrasystémique).

## Discussion

Le ventricule droit à double chambre (VDDC) est une forme de cardiopathie congénitale dans laquelle le ventricule droit est divisé en deux chambres (une chambre proximale à haute pression et une chambre distale à basse pression) par une bande musculaire anormale [1]. Il s'agit d'une forme rare de cardiopathie congénitale dont l'incidence varie entre 0,5 et 2% [2]. La plupart des cas de VDDC sont vus dans la petite enfance et parfois à l'adolescence entre 4 mois et 20 ans [1]. Rarement des cas asymptomatiques ou non diagnostiqués peuvent se révéler à l'âge adulte [1–4]. Chez cette patiente l'âge de présentation était de 16 ans, même si les symptômes évoluaient depuis l'enfance et auraient pu conduire à un diagnostic plus précoce. Les patients sont souvent asymptomatiques et sont référés pour l'évaluation d'un souffle. Dans une étude portant sur 52 patients, Cil et al. [1] rapportaient 40% de patients asymptomatiques, 35 % de patients avec asthénie, 17% avec une dyspnée d'effort tandis que l'insuffisance cardiaque, la cyanose et les palpitations étaient présents dans 10 à 12% des cas. La syncope, le vertige, la douleur thoracique et l'endocardite infectieuse sont rapportés par d'autres auteurs comme mode de présentation du VDDC chez l'adulte [5]. Cette patiente présentait plusieurs symptômes dont la dyspnée d'effort depuis l'enfance, mais c'est la survenue de syncope d'effort qui a été l'élément incitant à la demande de soins. Le mode de présentation clinique chez cette patiente avec syncope d'effort semble commun aux formes avec obstruction intraventriculaire droite importante et PVD supra systémique responsable d'un bas débit systémique à l'effort. Dans 80 à 90% des cas, le VDDC est associé à d'autres malformations cardiaques [6] dont la communication interventriculaire est la plus fréquente (CIV) [3, 7]. Les autres malformations associées rapportées sont: la sténose sous aortique, la sténose valvulaire pulmonaire, la tétralogie de Fallot, les anomalies de retour veineux pulmonaire et la transposition des gros vaisseaux. Cette patiente présentait une forme isolée de VDDC comme quelques séries rapportées dans la littérature [8]. La fréquence des malformations cardiaques associées, incitent à les rechercher systématiquement devant tout diagnostic de VDDC.

L'échocardiographie Döppler trans-thoracique (ETT) est le moyen diagnostic efficace pour le diagnostic du VDDC chez l'enfant. Elle se révèle néanmoins beaucoup moins précise chez l'adulte [9]. Comme chez cette patiente, plusieurs auteurs ont rapporté l'utilisation exclusive de l'ETT pour le diagnostic dans les proportions de 8,3 à 15,6% [9]. La faible performance diagnostique de l'ETT pour le diagnostic définitif du VDDC chez l'adulte s'expliquerait par la proximité de la voie de chasse droite par rapport au transducteur en précordial. Devant cette difficulté technique, il est important de considérer le VDDC comme diagnostic différentiel de l'hypertension artériel pulmonaire (HTAP), devant toute augmentation du gradient trans-tricuspidien sur le flux de l'insuffisance tricuspide, chez un adulte porteur de cardiopathie congénitale [3]. L'échographie trans-oesophagienne (ETO), l'imagerie par résonnance magnétique (IRM) et le cathétérisme cardiaque diagnostique peuvent être envisagés en deuxième intention. Le traitement de la plupart des formes de VDDC est chirurgical. La procédure chirurgicale consiste en une résection de la bande musculaire anormale et à la correction des anomalies cardiaques associées. En l'absence de défaut septal significatif associé, la stratégie est le suivi aussi longtemps que le gradient de pression intraventriculaire n'excède pas 40 mmHg et que l'obstruction ne progresse pas [5]. Chez cette patiente, la pression intraventriculaire droite était suprasystémique témoignant de la gravité de cette forme de VDDC. Le pronostic à long terme de la réparation chirurgicale du VDDC est excellent [10] mais ¾ des patients présentent un bloc de branche droit incomplet ou complet dans certaines séries [4]. Les résultats à court terme étaient excellents chez notre patiente. Un suivi est néanmoins indispensable pour apprécier le pronostic à moyen et à long terme. En effet, des patients ayant bénéficié d'une correction chirurgicale de VDDC ont eu besoin d'un traitement anti arythmique voire d'une thérapeutique ablative pour le traitement d'une tachycardie ventriculaire [4]. La récidive de VDDC après correction chirurgicale semble rare [10].

## Conclusion

Le ventricule droit à double chambre (VDDC) est une malformation congénitale rare, polymorphe dans sa présentation clinique. Il s'agit du premier cas diagnostiqué dans notre pratique au CNHU de Cotonou. L'échocardiographie Döppler reste un outil diagnostique performant chez l'enfant et l'adolescent. Le pronostic à court et à long terme est en général bon après résection chirurgicale de la bande musculaire anormale.

## Conflits d'intérêts

Les auteurs ne déclarent aucun conflit d'intérêts.
